# Genome Characteristics of the Endophytic Fungus *Talaromyces* sp. DC2 Isolated from *Catharanthus roseus* (L.) G. Don

**DOI:** 10.3390/jof10050352

**Published:** 2024-05-15

**Authors:** Nguyen Duc Quan, Ngoc-Lan Nguyen, Tran Thi Huong Giang, Nguyen Thi Thanh Ngan, Nguyen Thanh Hien, Nguyen Van Tung, Nguyen Hoang Thanh Trang, Nguyen Thi Kim Lien, Huy Hoang Nguyen

**Affiliations:** 1 Institute of Genome Research, Vietnam Academy of Science and Technology, 18 Hoang Quoc Viet Str., Cau Giay, Hanoi 100000, Vietnam; ducquan0709@gmail.com (N.D.Q.); lannguyen@igr.ac.vn (N.-L.N.); huonggiang.igr@gmail.com (T.T.H.G.); nganthanh27@yahoo.com (N.T.T.N.); hiennt@igr.ac.vn (N.T.H.); tungnv53@gmail.com (N.V.T.); thanhtrang13022000hy@gmail.com (N.H.T.T.); ntkimlienibt@gmail.com (N.T.K.L.); 2 Department of Biotechnology, Graduate University of Science and Technology, Vietnam Academy of Science and Technology, 18 Hoang Quoc Viet Str., Cau Giay, Hanoi 100000, Vietnam

**Keywords:** *Talaromyces*, DC2, genome sequencing, endophytic fungus, *Catharanthus roseus*, secondary metabolites, plant cell wall degradation, *DDC* gene

## Abstract

*Talaromyces* sp. DC2 is an endophytic fungus that was isolated from the stem of *Catharanthus roseus* (L.) G. Don in Hanoi, Vietnam and is capable of producing vinca alkaloids. This study utilizes the PacBio Sequel technology to completely sequence the whole genome of *Talaromyces* sp. DC2The genome study revealed that DC2 contains a total of 34.58 Mb spanned by 156 contigs, with a GC content of 46.5%. The identification and prediction of functional protein-coding genes, tRNA, and rRNA were comprehensively predicted and highly annotated using various BLAST databases, including non-redundant (Nr) protein sequence, Gene Ontology (GO), Kyoto Encyclopedia of Genes and Genomes (KEGG), Clusters of Orthologous Groups (COG), and Carbohydrate-Active Enzymes (CAZy) databases. The genome of DC2 has a total of 149, 227, 65, 153, 53, and 6 genes responsible for cellulose, hemicellulose, lignin, pectin, chitin, starch, and inulin degradation, respectively. The Antibiotics and Secondary Metabolites Analysis Shell (AntiSMASH) analyses revealed that strain DC2 possesses 20 biosynthetic gene clusters responsible for producing secondary metabolites. The strain DC2 has also been found to harbor the *DDC* gene encoding aromatic L-amino acid decarboxylase enzyme. Conclusively, this study has provided a comprehensive understanding of the processes involved in secondary metabolites and the ability of the *Talaromyces* sp. DC2 strain to degrade plant cell walls.

## 1. Introduction

*Catharanthus roseus* (L.) G. Don is a flowering plant species in the family *Apocynaceae* [1]. *C. roseus* is widely distributed in the regions of America, Africa, Asia, southern Europe, Australia, and Vietnam [2]. The plant’s secondary metabolites exhibit a diverse range of beneficial effects in combating various diseases (leukemia, various types of cancer) and illnesses (sore throat, fever, indigestion, septic wounds, diabetes) [1]. Moreover, the plant is highly valued in the field of medicine due to the existence of numerous alkaloids with pharmaceutical properties, such as vindoline, vinblastine, catharanthine, vincristine, ajmalicine, reserpine, serpentine, horhammericine, tabersonine, leurosine, and lochnerine [1]. Among these alkaloids, vincristine, vindesine, and vinblastine have been recognized for their anticancer properties [3]. However, the plant only produces a limited quantity of these beneficial alkaloids. Many research efforts have been undertaken to enhance the production of vinca alkaloids.

Endophytic fungi have gained increased attention for their capacity to produce vinca alkaloids, such as *Fusarium oxysporum* [4], *Talaromyces radius* CrP20 [5], *Curvularia verruculosa* [6], *Botryosphaeria laricina* strain CRS1 [7], and *Alternaria alternata* AUMC14391 [8]. Several studies on *C. roseus* have discovered that the utilization of biotic elicitors, such as fungal concentrate, can effectively increase the synthesis of secondary metabolites under in vitro conditions. Several endophytic fungal strains elicit the accumulation of vinca alkaloids in the leaves of *C. roseus*. Inoculation of *C. roseus* with endophytes (*Curvularia* sp. CATDLF5 and *Choanephora infundibulifera* CATDLF6) was found to enhance vindoline content. This was achieved by upregulating genes associated with the terpenoid indole alkaloid biosynthesis in *C. roseus* [9]. Previous research also found that cell extracts of endophytic fungi, such as *Fusarium solani* RN1 and *Chaetomium funicola* RN3, greatly increased the accumulation of alkaloids in the cell suspension culture system [10].

The *Talaromyces* is a genus of approximately 80 fungal species within the *Trichocomaceae* family; each of them has unique functions. Many of them can cause fungal pathogens like *Talaromyces marneffei*, *T. indigoticus*, *T. piceus*, *T. radicus*, *T. helicus*, *T. amestolkiae*, and *T. stollii* [11]. Several species can produce bioactive compounds, like *T. pinophilus*, *T. stipitatus*, *T. purpurogenus*, and *T. wortmannii* [12]. Other members have a great impact on the food industry [13]. For instance, *T. bacillisporus*, *T. flavus*, *T. helicus*, *T. macrosporus*, *T. stipitatus*, *T. trachyspermusi*, and *T. wortmannii* cause spoilage of pasteurized juices. *T. purpurogenus* produces mycotoxins. *T. islandicus* causes rice yellowing. The ability to produce enzymes and soluble pigments makes *Talaromyces* an important genus for biotechnological purposes. For example, *T. funiculosus* generates cellulase and utilizes it to produce ethanol through the hydrolysis of sugar cane bagasse [14]. *T. atroroseus* [15], *T. assiutensis* [16], and *T. albobiverticillius* [17] can synthesize pigments that have application as cosmetic and food colorants. In addition, the *Talaromyces* species are also capable of producing a wide range of secondary metabolites, including esters, coumarins, isocoumarin, polyketones, anthraquinone, terpenoids, meroterpenoids, steroids, alkaloids, and others [18].

Many studies have also revealed that *Talaromyces* species possess gene clusters associated with cell wall-degrading enzymes and secondary metabolites. Whole genome se quencing of strain *T. piceus* 9-3 revealed that its genome had a diverse set of lignocellulolytic enzymes, including two cellobiohydrolases, one endo-β-1,4-glucanase, and ten β-glucosidase gene clusters [19]. The genome of strain *T. pinophilus* 1–95 contained two cellobiohydrolases, eight β-1,4-endoglucanases, 29 β-glucosidases, 97 hemicellulose-degrading enzymes, 24 α-amylases, and 52 secondary metabolism gene clusters [20]. The genome of *T. albobiverticillius* Tp-2 contained eight distinct gene clusters responsible for the biosynthesis of secondary metabolites [17]. The genome of *T. albobiverticillius* Tp-2 contained eight distinct gene clusters responsible for the biosynthesis of secondary metabolites. In this study, the whole genome sequencing of the *Talaromyces* DC2 strain was performed using PacBio Sequel and Illumina NovaSeq 6000 sequencing platforms. The *Talaromyces* DC2 strain has already been identified by our research as a prolific producer of vinca alkaloid with anticancer properties [21]. The acquired whole genome sequencing data enrich our understanding of the relationships between gene clusters and metabolic products in the DC2 strain. Our results demonstrated that the DC2 strain has the capability to degrade pectin and starch, synthesize xylooligosaccharides and short-chain fructooligosaccharides, and produce swainsonine, varicidin A, asperterpenoid A, squalestatin S1, ustethylin A, and ilicicolin H, as well as perform the decarboxylation of L-tryptophan. Furthermore, the obtained whole genome data can serve as a valuable resource for future bioengineering research.

## 2. Materials and Methods

### 2.1. Fungal Strain

Strain DC2 was isolated from the surface sterilized stem of the *Catharanthus roseus* (L.) G. Don plant cultivated in Hanoi, Vietnam, with a yellow-colored colony as previously described [21]. The protocol for obtaining endophytic fungi from plant materials has been elucidated in a previous study [21]. Strain DC2 has already been proven to have the ability to produce anticancer compounds, including vincristine and vinblastine. Identification and quantification of vincristine and vinblastine produced by the DC2 strain were conducted by ultra-high performance liquid chromatography/multiple reaction monitoring mass spectrometry analyses.

### 2.2. Extraction of Genomic DNA

For DNA extraction, isolated endophytic fungi were inoculated in 100 mL potato dextrose broth (PDB; Sigma, Saint Louis, MO, USA) medium and cultured in 250 mL Erlenmeyer flasks at 25 °C in the dark on a rotary shaker at 200 rpm. After 7 days, the fungal biomass was harvested by centrifugation at 10,000 rpm for 15 min on an Eppendorf 5810R centrifuge (Eppendorf, Hamburg, Germany) and used for DNA extraction by the cetyltrimethylammonium bromide (CTAB) method with minor adjustments for optimization [22]. Qualification and quantification of extracted DNA were measured using a Nanodrop^®^1000 spectrophotometer (Thermo Scientific, Waltham, MA, USA).

### 2.3. Genome Sequencing

The genomic DNA of strain DC2 was sequenced using the PacBio Sequel system (Menlo Park, CA, USA) and the Illumina NovaSeq 6000 (San Diego, CA, USA). For the PacBio sequencing library, 5–10 µg of genomic DNA was sheared into 10–15 kb fragments using a g-TUBE device (PerkinElmer, Ho Chi Minh City, Vietnam). Then the library was constructed using the SMRTbell Express Template Preparation Kit 2.0 (Pacbio, Menlo Park, CA, USA), following the manufacturer’s protocol. In brief, the process involved amplifying the DNA fragments using barcoded DNA primers, resulting in a pooled collection of all the samples. For the Illumina sequencing library, the library was prepared using the VAHTS Universal Pro DNA Library Prep Kit (Vazyme, Nanjing, China) following the manufacturer’s protocol. The generated library was cleaned up, and the sequencing process was carried out using 2–150 paired-end (PE) and 10–15 kb read length configurations for Illumina and PacBio sequencing, respectively.

### 2.4. Assembly, Gene Prediction and Annotation

PacBio and Illumina reads were assembled using Hifiasm (v0.13-r308) and Canu (v1.7). The assembly result was corrected with Pilon (v1.22). The gene prediction was performed using Augustus (v3.3) with default parameters. The tRNAs, rRNAs, and non-coding RNAs were predicted using tRNA scan-SE (v1.3.1), barrnap (v0.9), and Rfam (v12.2) [23], respectively. The repeat sequences were detected using RepeatMasker (v4.0.6) using the Dfam database (v2.0) (http://www.repeatmasker.org, accessed on 6 December 2023).

BLAST searches of non-redundant (NR) protein sequences from the NCBI, Kyoto Encyclopedia of Genes and Genomes (KEGG) [24], Gene Ontology (GO) [25], Clusters of Orthologous Groups (COG/KOG) [26], Carbohydrate-Active Enzymes (CAZy), Pfam, Swiss-Prot, and Database of Fungal Virulence Factors (DFVF) databases were performed to annotate the gene products. Signal peptides were analyzed using the online software SignalP v.5.0 (http://www.cbs.dtu.dk/services/SignalP/, accessed on 6 December 2023). The polypeptide chain of a transmembrane protein was analyzed using the online software TMHMM v.2.0 (http://www.cbs.dtu.dk/services/TMHMM/, accessed on 6 December 2023).

Secondary metabolite biosynthetic clusters were identified using the antiSMASH web server (fungal version 7.0.1) with the default settings [27].

## 3. Results and Discussion

### 3.1. Genome Sequencing, Assembly, and Genomic Features

The Illumina sequencing data yielded a total of 39,360,260 clean reads, which corresponds to 5,900,310,550 bases. These readings had 91.83% of their bases with a quality score of Q30. On the other hand, the PacBio sequencing data produced 107,913 raw reads, totaling 340,232,173 bases. The N50 value was 3423 bp. After assembly, a total of 156 scaffolds were obtained, with a total size of 34,575,287 bp (Table 1). The final assembly revealed a GC content of 45.94%. The genome size of strain DC2 was compared to the recently available 75 genome sizes in NCBI, which range from 26.6 Mb of *Talaromyces piceae* strain 9-3 (GCA_001657655.1) to 42.5 Mb of *Talaromyces nanjingensis* strain JP-NJ4 (GCA_031010415.1) (Supplementary Table S1).

### 3.2. The Gene Functions of Talaromyces sp. DC2

A total of 11,131 genes were annotated in the genome of strain DC2 (Table 1). The highest number of functional genes in DC2 was determined by the NR database (10,780 genes, 96.85%), followed by Pfam (8735 genes, 78.47%), Swiss-Prot (8165 genes, 73.35%), GO (6682 genes, 60.03%), KEGG (6509 genes, 58.48%), KOG (6353 genes, 57.07%), DFVF (2658 genes, 23.88%), and CAZy (1230 genes, 11.05%) (Table 1). The number of protein-coding genes in DC2 is lower than that of *T. pinophilus* 1-95, which consisted of 13,472 protein-coding genes [20], but higher than that of *T. albobiverticillius* Tp-2, which consisted of 10,380 protein-coding genes [17]. Variations in the number of protein-coding genes may result from variations in the quality of the input DNA quality, the sequencing technique employed, and/or the size and native sequence of the genome. 

**Figure 1:**
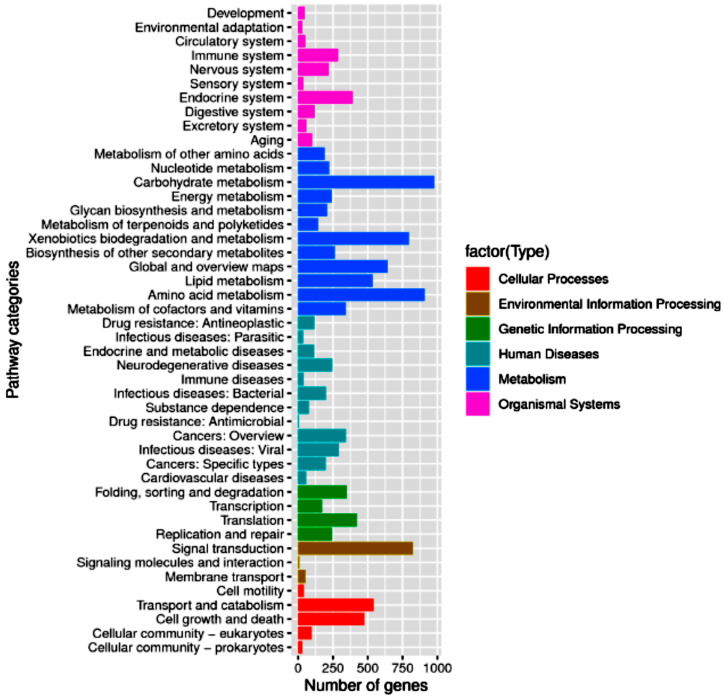
Kyoto Encyclopedia of Genes and Genomes (KEGG) functional classification of *Talaromyces* sp. DC2.

The 6509 genes were mapped to known enzyme pathways in six KEGG types: cellular processes, environmental information processing, genetic information processing, human diseases, metabolism, and organismal systems (Figure 1). The most abundant pathways in DC2 include carbohydrate metabolism (978), amino acid metabolism (908), signal transduction (824), and xenobiotics biodegradation and metabolism (796). The abundance of genes in the xenobiotics biodegradation and metabolism, as well as signal transduction pathways, suggests that strain DC2 is capable of metabolizing xenobiotics in its environments.

### 3.3. Carbohydrate-Active Enzymes in Talaromyces sp. DC2

**Figure 2:**
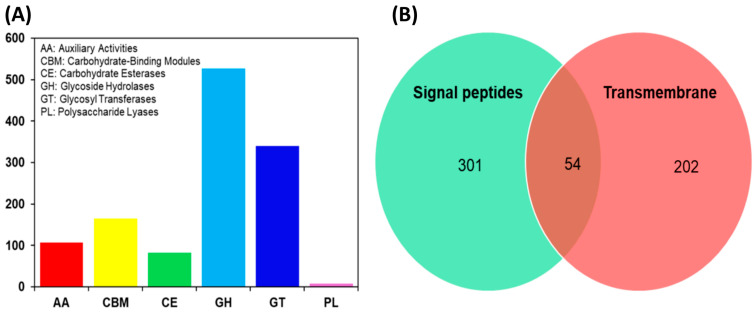
Distribution of carbohydrate-active enzyme (CAZyme) familes (**A**), and signal and transmembrane CAZymes (**B**) in *Talaromyces* sp. DC2.

Carbohydrate-active enzymes (CAZymes) play important roles in carbohydrate degradation. We detected a total of 1230 genes classified as CAZymes in strain DC2. The total number of CAZymes in DC2 is greater than those of other *Talaromyces* strains, for example, *T. pinophilus* strain 1–95 (803 CAZymes) [20] and *T. albobiverticillius* strain Tp-2 (750 CAZymes) [17]. DC2 possesses a total of 527 glycoside hydrolases (GHs), 340 glycosyltransferases (GTs), 165 carbohydrate-binding modules (CBMs), 107 auxiliary activities (AAs), 83 carbohydrate esterases (CEs), and 8 polysaccharide lyases (PLs) (Figure 2A). The number of GH family genes in *Talaromyces* sp. DC2 (527 genes) is higher than those of *T. albobiverticillius* strain Tp-2 (427 genes) [17] and *T. cellulolyticus* (249 genes) [28]. GHs accounted for 42.85% of the CAZymes and were found in 74 families. The most prevalent families were GH18 (chitinase) with 56 out of 527 members and GH43 (β-xylosidase) with 36 out of 527 members. GTs comprised 43 families, consisting of 74 cellulose synthases from the GT2 family and 52 sucrose synthases from the GT4 family. CBMs included 25 families, with the highest prevalence found in family XIII of the cellulose-binding domain, accounting for 37 out of 165 members. The primary families of AAs were predominantly AA3 (34 out of 107), AA7 (28 out of 107), and AA1 (15 out of 107) families. The CEs were categorized into families of CE0-CE6, CE8-9, CE11-12, and CE14-CE16. PLs were distributed in PL0-PL1, PL4, PL7, and PL10.

In DC2, 507 out of 1230 CAZymes (41.22%) contained a signal peptide and/or transmembrane (Figure 2B and Supplementary Table S1). There are 54 CAZymes that have both signal peptides and transmembrane domains. Additionally, there are 301 CAZymes that have signal peptides but do not have transmembrane domains, and 202 CAZymes that have transmembrane domains but lack signal peptides. The 301 CAZymes possessing signal peptides but lacking transmembrane domains were classified as secreted CAZymes. It was found that the DC2 strain secrets a similar number of CAZymes to *T. pinophilus* strain 1–95, specifically, 323 CAZymes [20].

Strain DC2 was isolated from the stem of *C. roseus* [21]; therefore, we focused on plant cell wall-degrading CAZymes as described in previous studies [29,30]. Strain DC2 possess 653 CAZymes responsible for plant cell wall degradation. They are involved in degradation of cellulose (149 CAZymes), hemicellulose (227 CAZymes), lignin (65 CAZymes), pectin (153 CAZymes), starch (53 CAZymes), and inulin (6 CAZymes) (Table 2). Of these, β-glucosidases (27 genes) were involved in both cellulose and hemicellulose degradations; β-galactosidases (23 genes) and α-L-arabinofuranosidases (6 genes) were involved in both hemicellulose and pectin degradations (Table 2). The plant cell wall-degrading CAZymes of strain DC2 have been found to exhibit greater diversity compared to those of *T. pinophilus* strain 1–95 [20].

Cellulose is one of the three most abundant polysaccharides in plant cell walls and has a basic structure of β-1,4-linked D-glucose molecules [34]. Cellulose degradation involves the activities of endocellulases, exoglucanases, cellobiohydrolases, and β-glucosidases [35]. Strain DC2 consisted of 6 genes encoding exoglucanases (also known as cellobiohydrolases) (GH55), 43 genes encoding endoglucanases, 27 genes encoding β-glucosidases, and 3 cellobiose dehydrogenases. However, it did not have a gene encoding lytic polysaccharide monoxygenase (LPMO) (Table 2). Other fungal strains, in contrast, have a different number of genes encoding cellulose-degrading enzymes. Specifically, *Trichoderma reesei* QM6a is the only strain that harbors six genes encoding exoglucanases (GH23: two genes and GH55: four genes) [32], while information regarding the exoglucanases of the remaining five fungal strains is not available. In endoglucanases, there are 18 genes in the *T. reesei* QM6a, 9 genes in the *Aspergillus niger* CBS 513.88, 14 genes in the *T. amestolkiae* CIB, 10 genes in the *T. pinophilus* 1–95, 9 genes in the *T. cellulolyticus* Y-94, and 12 genes in the *Penicillium oxalicum* HP7-1 (Table 2). In β-glucosidase, the *T. reesei* QM6a, *A. niger* CBS 513.88, *T. pinophilus* 1–95, *T. cellulolyticus* Y-94, and *P. oxalicum* HP7-1 strains consist of 13, 18, 24, 19, and 8 genes, respectively (Table 2), while LPMO has only been found in the *T. amestolkiae* CIB (one gene), *T. pinophilus* 1–95 (one gene), *T. cellulolyticus* Y-94 (one gene), and *P. oxalicum* HP7-1 strains (three genes). Polysaccharide monooxygenase has only been found in the *T. reesei* QM6a (three genes) and *A. niger* CBS 513.88 (seven genes). The data suggest that the *Talaromyces* DC2 strain and *T. reesei* QM6a are the only two strains that have a nearly complete set of enzymes for effectively degrading cellulose.

Hemicellulose is composed of β-1,4-xylan and β-1,4-D-xylopyranoside [36]. Xylan degradation depends primarily on two enzymes, endo-β-1,4-xylanase and β-1,4-D-xylosidase [37]. In strain DC2, one endo-β-1,4-xylanase family (GH10) and one β-xylosidase family (GH43) were detected (Table 2). There are 36 genes encoding β-xylosidases. Furthermore, strain DC2 also contained various side-chain cleaving hydrolases, such as α-L-arabinofuranosidases (6 genes), α-glucuronidases (1 gene), and acetyl xylan esterases (42 genes) (Table 2). Similarly, the six comparative fungal strains also harbor a range of genes encoding hemicellulose-degrading enzymes. Specifically, the *T. reesei* QM6a, *A. niger* CBS 513.88, *T. amestolkiae* CIB, *T. pinophilus* 1–95, *T. cellulolyticus* Y-94, and *P. oxalicum* HP7-1 strains consist of 6, 4, 16, 9, 7, and 10 endo-1,4-β-xylanase-encoding genes, respectively, as well as 3, 10, 16, 10, 7, and 12 genes encoding β-xylosidase, respectively. The results support the high xylanase activity of strain DC2, which is comparable to that of *T. pinophilus* 1–95 [20]. The byproducts of xylan breakdown include xylo-oligosaccharides and xylose, which have potential applications in several industries such as health care, food, pharmaceuticals, and cosmetics [38]. Thus, it is evident that strain DC2 shows potential as a valuable source for the synthesis of xylo-oligosaccharides, a potential material that has many applications in different industries.

Lignin is a complex polymer that embeds in cellulose and hemicellulose to strengthen the structure of the plant cell wall. The primary enzymes involved in lignin degradation are the laccase and peroxidase families [39]. Our data analysis showed that the DC2 strain contains 15 genes that encode laccase and 1 gene that encodes peroxidase. This suggested that that the DC2 strain has the potential to break down the lignin matrix (Table 2). The six fungal strains, interestingly, do not possess any genes that encode liginiolytic enzymes.

Pectin is a heteropolysaccharide abundant component of the plant’s primary cell wall [40]. Pectin consists of α-1,4-linked D-galacturonic acid and several sugars such as rhamnose, arabinose, galactose, and other sugars [41,42]. Strain DC2 consisted of a complete set of CAZymes for pectin degradation, including glycoside hydrolases (GH2, GH28, GH35, GH51, GH53, GH54, GH78, GH88, GH93, and GH105), polysaccharide lyases (PL1, PL4, PL7, and PL20), carbohydrate esterases (CE8, CE12, and CE16), and carbohydrate-binding modules (CBM13, CBM32, and CMB51) (Table 2). Strain DC2 exhibits a pectin degradation profile that is comparable to that of *T. pinophilus* 1–95, *T. cellulolyticus* Y-94 and *P. oxalicum* HP7-1 strains but more diverse than that of the *T. reesei* QM6a, *A. niger* CBS 513.88, and *T. amestolkiae* CIB (Table 2). As suggested by Benoit et al. [43], the increase in pectin-related genes resulted in improved growth in pectin. This suggests that strain DC2 has the ability to thrive on different pectin sources, such as citrus pectin and apple pectin. Thus, strain DC2 exhibits significant promise for the breakdown of pectin.

Starch is an α-1,4-linked D-glucose polymer that is synthesized by plants as a means of energy storage [44]. Strain DC2 consists of 13 GH13 genes encoding α-amylases, 2 GH15 genes encoding glucoamylases, 15 GH31 genes encoding α-glucosidase, 1 GT35 gene encoding starch phosphorylase (1 gene), and 21 genes encoding starch-binding (18 CBM20 and 3 CBM21) (Table 2). Comparative analysis revealed that only *T. reesei* QM6a and *A. niger* CBS 513.88 contain genes encoding starch-degrading enzymes, while these genes were not found in the remaining four fungal strains. Furthermore, our study revealed that strain DC2 possesses a higher number of genes that encode α-amylases and α-glucosidase than *T. pinophilus* 1–95 [20] and 85 fungal strains belonging to the phyla Ascomycota, Basidiomycota, Chytridiomycota, and Zygomycota [45].

Inulin is a fructan polysaccharide found in plants that serves as a storage carbohydrate. It consists of glucose molecules at the terminal end [46]. The process of inulin conversion involves the utilization of the glycosyl hydrolase families GH32 and GH91, which include enzymes such as inulinase, invertase, levanase, 1-exohydrolases, fructan-fructosyltransferases, and sucrose fructosyltransferases [47]. The genome of strain DC2 contained five genes encoding endo-inulinase from the GH32 family, which is consistent with the discovery made in *Apgergillus niger* [48]. Endo-inulinase breaks out the glycosidic bond β(2→1) to produce short-chain fructooligosaccharides [49]. Furthermore, strain DC2 was found to contain a gene encoding the inulin binding domain, CBM38. The data suggest that strain DC2 is capable of degrading inulin as an endobiont. In addition, the short-chain fructooligosaccharides serve as prebiotics [49]. Therefore, strain DC2 has the potential to be a great source for producing fructooligosaccharides that are similar to those produced by different *Aspergillus* strains [33,50,51]. 

### 3.4. The Secondary Metabolism in the DC2

AntiSMASH analysis suggested that strain DC2 possessed 37 biosynthetic gene clusters (BGCs) related to secondary metabolism. Among them, 20 out of 37 genes exhibited gene homologies with known clusters in the MIBiG database. These clusters included 12 Type I polyketide synthases (T1PKSs), five nonribosomal peptide synthetases (NRPSs and NRPS-like), and three terpene synthases (Terpenes) (Table 3). Strain DC2 has a lesser number of secondary metabolism BCGs compared to *T. pinophilus* strain 1-95 (68 clusters) [20] and *T. albobiverticillius* strain Tp-2 (62 clusters) [17].

There are six secondary metabolism clusters that have a gene similarity of 100% with six known biosynthetic clusters. These known clusters produce substances such as monascorubrin, YWA1, alternariol, ochratoxin, choline, and cyclic depsipeptide (Table 3). In region 9.1, one T1PKS was responsible for the biosynthesis of monascorubrin. This compound has been used as a natural red colorant for a wide range of foods in Asian countries [52]. Monascrorubin has been identified in *Talaromyces* species such as *T. marneffei* [52] and *T. atroroseus* [53]. In region 29.1, one T1PKS has been found to be responsible for the biosynthesis of naphthopyrone YWA1. This compound is considered a precursor of dihydroxynaphthalene (DHN)-melanin in *Aspergillus nidulans* [54] and aurofusarin in *Fusarium graminearum* [55]. In region 30.2, one T1PKS was found to be accountable for production of alternariol, a toxic metabolite in *Alternaria* that showed multiple potential pharmacological effects [30]. The alternariol has also been identified in *T. pinophilus* AF-02 [56]. Region 34.1 was responsible for the biosynthesis of ochratoxin A, a potent pentaketide nephrotoxin produced by *Aspergillus* and *Penicillium* species. This toxin can be detected in fungal contaminated food, beverages, and feed [57]. However, ochratoxin A was not included in the list of 238 secondary metabolite substances produced by *Talaromyces* species [18]. In region 43.2, one NRPS-like was responsible for the biosynthesis of choline, which is an essential metabolite for the growth of filamentous fungi and the regulation of mycelial morphology [58]. In region 69.1, one NRPS was identified as the catalyst for the production of cyclic depsipeptide. This occurs when the amide groups in the peptide structure are substituted with lactone bonds, which is facilitated by the presence of a hydroxylated carboxylic acid [59]. Cyclic peptides were discovered in *T. wortmannii* [60].

**Figure 3:**
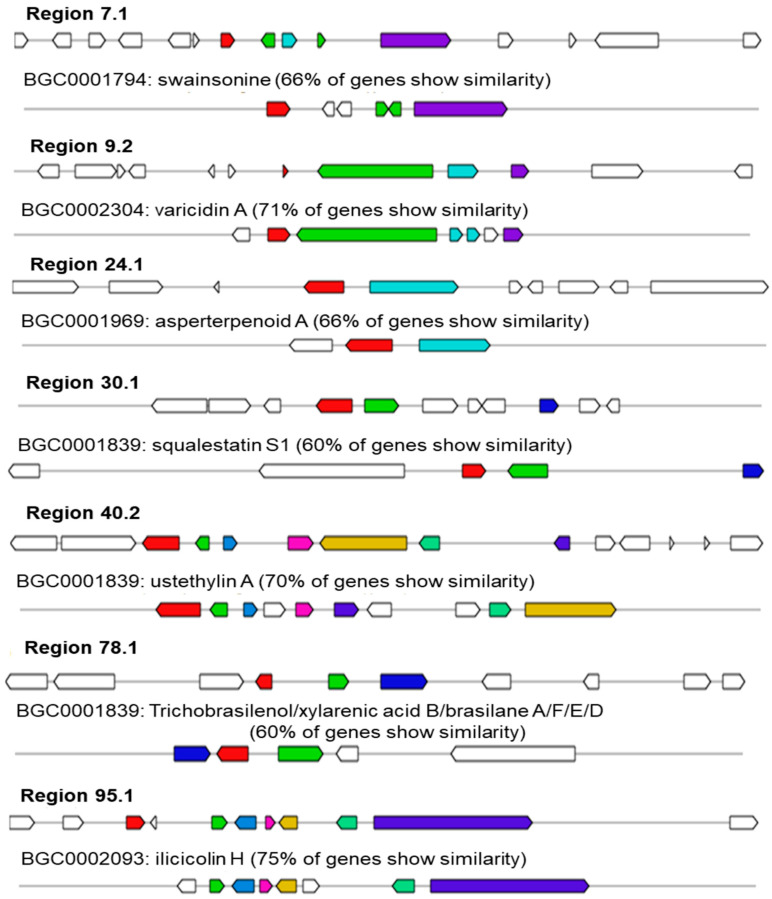
Comparison of biosynthetic gene cluster constituents in strain DC2 with identified biosynthetic gene clusters for biosynthesis of swainsonine (66%), varicidin A, asperterpenoid A, squalestatin S1, ustethylin A, trichobrasilenol/xylarenic acid B/brasilane A/F/E/D, and ilicicolin H.

Seven BGC clusters exhibit the similarities ranging from 60% to 75%, including swainsonine (66%), varicidin A (71%), asperterpenoid A (66%), squalestatin S1 (60%), ustethylin A (70%), trichobrasilenol/xylarenic acid B/brasilane A/F/E/D (60%), and ilicicolin H (75%) (Figure 3). In region 7.1, one T1PKS was responsible for the biosynthesis of swainsonine, an indolizidine alkaloid that is produced by endophytic fungi and has the potential to be used as a drug for cancer therapy [61,62]. In region 9.2, a single NRPS was found to be linked to the production of varicidin A. Varicidin A is an naturally occurring antifungal compound that is produced by a Diels–Alderase reaction [63]. In region 24.1, a single terpene was responsible for the production of asperterpenoid A, a compound which exhibits strong inhibitory activity against *Mycobacterium tuberculosis* protein tyrosine phosphatase B [64,65,66]. In region 30.1, a specific terpene was found to be accountable for the biosynthesis of squalestatin S1, which acts as a highly potent picomolar inhibitor of squalene synthase [67]. Additionally, squalestatin S1 exhibits a wide range of antifungal properties and serves as a lead structure for the development of cholesterol–lowering drugs [68]. In region 40.2, one T1PKS was responsible for the biosynthesis of ustethylin A, a compound synthesized by *Aspergillus ustus* [69]. In region 78.1, a specific terpene was identified to be responsible for the biosynthesis of trichobrasilenol/xylarenic acid B/brasilane A/F/E/D. This is an unusual sesquiterpene alcohol synthesized by a sesquiterpene cyclase from *Trichoderma* sp. [70]. In region 95.1, one NRPS was found to be accountable for the biosynthesis of ilicicolin H. This compound is a broad-spectrum antifungal agent that acts on mitochondrial cytochrome bc1 reductase [71,72,73,74,75]. This study, to our knowledge, is one of the first to report the presence of BGC clusters that encode swainsonine, varicidin A, asperterpenoid A, squalestatin S1, ustethylin A, trichobrasilenol/xylarenic acid B/brasilane A/F/E/D, and ilicicolin H in the *Talaromyces* genus.

### 3.5. The Indole Alkaloid Biosynthesis in the DC2

**Figure 4:**
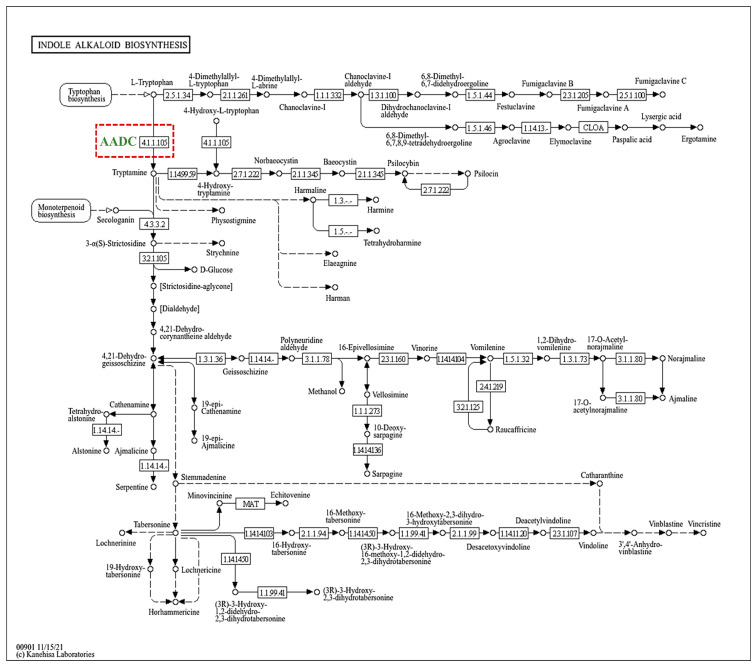
The indole alkaloid biosynthetic pathway gene found in *Talaromyces* sp. DC2 is depicted by the red-colored box. AADC, aromatic L-amino acid decarboxylase.

Decarboxylation of L-tryptophan leads to the formation of tryptamine, which serves as a common backbone for many secondary metabolites. One such metabolite is the pathway of terpenoid indole alkaloids in plants [76]. In strain DC2, a *DDC* gene (Gene ID: g533) that encodes an aromatic L-amino acid decarboxylase (AADC) was identified (Figure 4). 

**Figure 5:**
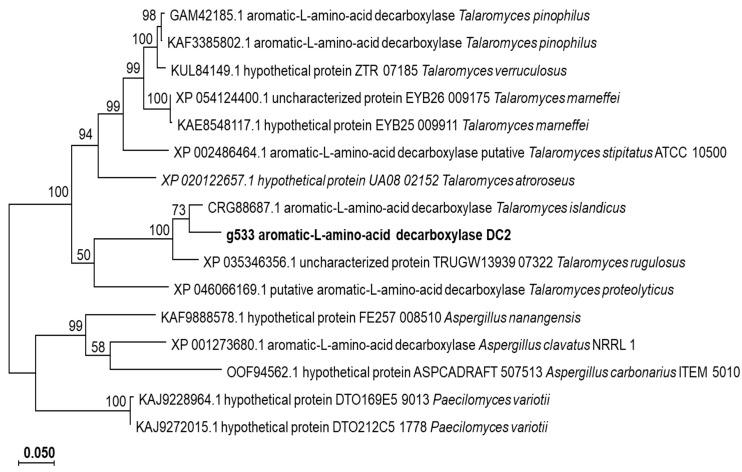
Phylogenetic tree includes the amino acid sequences of putative aromatic L-amino acid decarboxylases. MUSCLE v.5.0 was used for sequence alignment and a neighbor-joining algorithm was used to construct the tree in the Mega-X v.10.2.6.

The open reading frame (ORF) of g533 had a length of 1536 nucleotides and corresponded to the coding sequence for 512 amino acids (Supplementary Figure S1). The g533 shared the closest genetic similarities to those of *Talaromyces islandicus* (CRG88687.1; 93.58%) and *Talaromyces rugulosus7* (XP_035346356.1; 90.91%). The three sequences also formed a clade in the phylogenetic gene (Figure 5).

In contrast to AADCs from animals and plants, fungal AADCs have not been extensively studied. The first description of a fungal AADC was reported by Niedens et al. [77]. The authors demonstrated its broad substrate specificity, including L-tryptophan, L-tyrosine, L-phenylalanine, o-fluorophenylalanine, and p-fluorophenylalanine. Later, Kalb et al. [78] reported on the *Ceriporiopsis subvermispora* aromatic L-amino acid decarboxylases (*C*sTDCs) that were heterologously produced in a laboratory setting. The study identified that *Cs*TDC exhibited strict specificity towards L-tryptophan and 5-hydroxy-L-tryptophan. Interestingly, AADC of strain DC2 in our study also contains the same sequence, ^368^LGRRFR^373^, as *C*sTDC’s sequence, ^350^LGRRFR^355^, where G351 is the active site. However, *C*sTDC has a phenylalanine at residue 329, whereas that of g533 has tyrosine. This is similar to the amino acid sequence of PcDHPAAS, which is capable of converting L-3,4-dihydroxyphenylalanin to 3,4-dihydroxylphenylacetaldehyde [79]. However, further investigation is required to assess the decarboxylation capacity of g533 towards aromatic amino acids.

## 4. Conclusions

In summary, whole genome sequencing has provided a comprehensive understanding of *Talaromyces* sp. DC2, encompassing its overall functions of CAZymes and secondary metabolites. Genome analysis showed that strain DC2 might serve as a potential source for the degradation of pectin and starch, the synthesis of xylo-oligosaccharides and short-chain fructooligosaccharides, and the production of swainsonine, varicidin A, asperterpenoid A, squalestatin S1, ustethylin A, and ilicicolin H. Additionally, it has the ability to carry out the fungal decarboxylation of L-tryptophan. Furthermore, the obtained genome sequencing data can serve as a valuable resource for future bioengineering research. However, further investigations are required to confirm the distinct characteristics and feasibility of the *Talaromyces* DC2 strain.

## Figures and Tables

**Table 1:** Genome summary statistics for *Talaromyces* sp. DC2 and related strains.

Characteristics	*Talaromyces* sp. DC2		*Talaromyces pinophilus* 1–95 [20]	*Talaromyces albobiverticillius* Tp-2 [17]
	Value	% of Total		
Genome assembly (bp)	34,575,287	100%	36,480,443	38,354,882
Contigs	156	-	1	14
N50 length (bp)	346,458	-	4,804,168	4,594,200
Minimum length (bp)	3491	-	2,941,929	-
Maximum length (bp)	1,007,571	-	7,684,667	6,575,826
G+C content	15,883,165 bp	45.94%	46.25%	45.78%
Coding region	17,667,222 bp	51.10	-	-
Total genes	11,131	100	13,579	10,584
RNA genes	142	1.28	107	204
Protein-coding genes	10,989	98.72	13,472	10,380
NR	10,780	96.85	12,946	9782
KEGG	6509	58.48	6817	8844
GO	6682	60.03	8162	7412
KOG	6353	57.07	-	2160
CAZy	1230	11.05	803	750
Pfam	8735	78.47	-	7412
Swiss-Prot	8165	73.35	-	3657
DFVF	2658	23.88	-	2058

NR, non-redundant; KEGG, Kyoto Encyclopedia of Genes and Genomes; GO, Gene Ontology; KOG, Clusters of Orthologous Groups; CAZy, carbohydrate-active enzymes; DFVF, Database of Fungal Virulence Factors.

**Table 2:** Plant cell wall-degrading carbohydrate-active enzymes (CAZymes) in *Talaromyces* sp. DC2 and other fungal strains.

Substrate	Enzymatic Activity	CAZy Family	*Talaromyces* sp. DC2	*T. pinophilus* 1-95 [20]	*T*. *cellulolyticus* Y-94 [20]	*T. amestolkiae* CIB [31]	*Trichoderma reesei* QM6a [32]	*Aspergillus niger* CBS 513.88 [33]	*Penicillium oxalicum* HP7-1 [20]
Cellulose	Endoglucanase	GH5	17	4	4	14	4	3	5
		GH6	1	1	0	n/a	1	2	1
		GH7	2	1	1	n/a	2	2	2
		GH12	3	2	2	n/a	2	2	3
		GH45	1	2	2	n/a	0	0	1
		GH64	5	n/a	n/a	n/a	3	0	n/a
		GH71	11	n/a	n/a	n/a	4	0	n/a
		GH81	2	n/a	n/a	n/a	2	0	n/a
		GH131	1	n/a	n/a	n/a	0	0	n/a
	Exoglucanase	GH23	0	n/a	n/a	n/a	2	0	n/a
		GH55	6	n/a	n/a	n/a	4	0	n/a
	β-glucosidase	GH1	3	5	5	n/a	2	3	4
		GH3	24	24	19	n/a	11	15	8
	Polysaccharide monooxygenase	GH61	0	n/a	n/a	n/a	3	7	n/a
	Cellulose-binding	CBM1	11	n/a	n/a	n/a	n/a	n/a	n/a
		CBM2	18	n/a	n/a	n/a	n/a	n/a	n/a
		CBM3	1	n/a	n/a	n/a	n/a	n/a	n/a
		CBM6	5	n/a	n/a	n/a	n/a	n/a	n/a
		CBM10	1	n/a	n/a	n/a	n/a	n/a	n/a
	Lytic polysaccharide monoxygenase (LPMO)	AA16	0	n/a	n/a	n/a	n/a	n/a	n/a
		AA9	0	1	1	1	n/a	n/a	3
	Cellobiose dehydrogenase	AA8	3	n/a	n/a	n/a	n/a	n/a	n/a
	FAD-dependent (GMC) oxidoreductase	AA3	34	n/a	n/a	n/a	n/a	n/a	n/a
Hemicellulose	Endo-1,4-β-xylanase	GH10	1	1	1	1	1	1	3
		GH11	3	8	6	9	3	3	5
		GH30	0	n/a	n/a	6	2	0	2
	Xyloglucanase	GH74	0	n/a	n/a	3	1	1	n/a
	β-xylosidase	GH43	36	10	7	16	3	10	12
	β-glucosidase	GH1	3	5	5	n/a	2	3	4
		GH3	24	24	19	n/a	11	15	8
	α-mannosidase	GH47	18	n/a	n/a	n/a	8	0	n/a
		GH92	0	n/a	n/a	n/a	6	0	n/a
	α-L-arabinofuranosidase	GH51	2	3	3	n/a	0	3	3
		GH54	4	5	5	n/a	2	1	1
		GH62	0	3	3	3	1	1	2
	α -1,6-mannanase	GH76	21	n/a	n/a	n/a	7	0	n/a
	Β-mannanase	GH26	0	n/a	n/a	n/a	n/a	1	1
	Endo-α-1,5-arabinanase	GH93	14	4	3	n/a	0	0	3
	β-glucuronidase	GH79	7	n/a	n/a	n/a	4	0	n/a
	α-glucuronidase	GH67	1	n/a	n/a	1	1	1	1
	α-galactosidase	GH27	9	4	4	n/a	8	5	2
		GH36	12	4	3	n/a	2	2	n/a
	β-galactosidase	GH2	13	6	6	n/a	6	4	2
		GH35	10	n/a	n/a	n/a	1	5	n/a
	α-L-fucosidase	GH29	1	4	3	n/a	0	1	n/a
		GH95	1	5	3	n/a	3	2	1
	Acetyl xylan esterase	CE1	7	2	2	1	n/a	n/a	1
		CE2	1	2	2	n/a	n/a	n/a	1
		CE3	3	7	5	n/a	n/a	n/a	n/a
		CE4	7	n/a	n/a	n/a	n/a	n/a	n/a
		CE5	5	2	1	3	n/a	n/a	1
		CE6	19	n/a	n/a	n/a	n/a	n/a	n/a
	Arabinoxylan-binding	CBM42	5	n/a	n/a	n/a	n/a	n/a	n/a
Lignin	Laccase	AA1	15	n/a	n/a	n/a	n/a	n/a	n/a
	Peroxidase	AA2	1	n/a	n/a	n/a	n/a	n/a	n/a
	FAD-dependent (GMC) oxidoreductase	AA3	34	n/a	n/a	n/a	n/a	n/a	n/a
	Vanillin alcohol oxidase	AA4	6	n/a	n/a	n/a	n/a	n/a	n/a
	Copper radical oxidase	AA5	3	n/a	n/a	n/a	n/a	n/a	n/a
	Benzoquinon reductase	AA6	2	n/a	n/a	n/a	n/a	n/a	n/a
	Cellobiose dehydrogenase	AA8	3	n/a	n/a	n/a	n/a	n/a	n/a
	4-O-methyl-glucuronoyl methylesterase	CE15	1	n/a	n/a	n/a	n/a	0	n/a
Pectin	Polygalacturonase	GH28	22	3	2	n/a	4	21	8
	α-L-arabinofuranosidase	GH51	2	3	3	n/a	0	0	3
		GH54	4	5	5	n/a	2	0	1
	Exo-α-L-1,5-arabinanase	GH93	14	4	3	n/a	0	0	3
	β-galactosidase	GH2	13	6	6	n/a	6	4	2
		GH35	10	n/a	n/a	n/a	1	5	n/a
	d-4,5 unsaturated β-glucuronyl hydrolase	GH88	3	n/a	n/a	n/a	0	0	1
	unsaturated rhamnogalacturonyl hydrolase	GH105	2	4	4	n/a	1	0	1
	α-L-rhamnosidase	GH78	17	n/a	n/a	n/a	1	8	n/a
	β-1,4-galactanase	GH53	1	1	1	n/a	0	0	1
	Pectate lyase	PL1	1	2	2	n/a	n/a	6	1
	Rhamnogalacturonan lyase	PL4	1	n/a	n/a	n/a	n/a	2	1
	α-L-guluronate lyase	PL7	1	n/a	n/a	n/a	n/a	0	n/a
	Endo-β-1,4-glucuronan lyase	PL20	1	n/a	n/a	n/a	n/a	0	n/a
	Pectin methylesterase	CE8	4	3	3	n/a	n/a	3	3
	Pectin acetylesterase	CE12	2	1	1	n/a	n/a	2	2
	Acetylesterase	CE16	7	n/a	n/a	n/a	n/a	0	n/a
	Galactose-binding (48 genes)	CBM13	37	n/a	n/a	n/a	n/a	n/a	n/a
		CBM32	9	n/a	n/a	n/a	n/a	n/a	n/a
		CMB51	2	n/a	n/a	n/a	n/a	n/a	n/a
Starch	α-amylase	GH13	13	n/a	n/a	n/a	1	7	n/a
	Glucoamylase	GH15	2	n/a	n/a	n/a	2	1	n/a
	α-glucosidase	GH31	15	n/a	n/a	n/a	4	7	n/a
		GH63	1	n/a	n/a	n/a	2	0	n/a
	Starch phosphorylase	GT35	1	n/a	n/a	n/a	n/a	n/a	n/a
	Starch-binding	CBM20	18	n/a	n/a	n/a	n/a	n/a	n/a
		CBM21	3	n/a	n/a	n/a	n/a	n/a	n/a
Inulin	Endo-inulinase	GH32	5	n/a	n/a	n/a	n/a	1	n/a
	Inulin-binding	CBM38	1	n/a	n/a	n/a	n/a	n/a	n/a

n/a: not applicable.

**Table 3:** Putative biosynthetic gene clusters of *Talaromyces* sp. DC2 showed similarity to known gene clusters in the minimum information about a biosynthetic gene cluster database.

No.	Region	Type	Location	Most Similar Known Cluster	Similarity
1	7.1	T1PKS	309,280–374,892	Swainsonine	66%
2	9.1	T1PKS	4143–41,865	Monascorubrin	100%
3	9.2	NRPS	142,422–212,589	Varicidin A	71%
4	22.1	T1PKS	296,509–349,694	Etanone C/probetaenone	42%
5	24.1	Terpene	236,960–270,368	Asperterpenoid A	66%
6	29.1	T1PKS	69,255–135,872	YWA1	100%
7	30.1	Terpene	76,815–108,403	Squalestatin S1	60%
8	30.2	T1PKS	219,673–284,128	Alternariol	100%
9	34.1	T1PKS	81,492–145,061	Ochratoxin	100%
10	40.1	T1PKS	526,164–588,670	Waikikiamide A/B/C	18%
11	40.2	T1PKS	601,377–668,240	Ustethylin A	70%
12	43.2	NRPS-like	181,715–301,302	Choline	100%
13	52.1	T1PKS	183,397–251,045	Waikikiamide A/B/C	36%
14	61.1	T1PKS	194,350–261,094	3’-methoxy-1,2-dehydropenicillide/pestalotiollide B/C	10%
15	63.1	T1PKS	32,753–101,221	Gregatin	33%
16	65.1	T1PKS	71,173–135,718	Crytosporioptide B/A/C	15%
17	69.1	NRPS	40,492–110,190	Cyclic depsipeptide	100%
18	78.1	Terpene	200,100–231,945	Trichobrasilenol/xylarenic acid B/brasilane A/F/E/D	60%
19	93.1	NRPS	1–41,092	Dihydroisoflavipucine/isoflavipucine	31%
20	95.1	NRPS	246,099–307,834	Ilicicolin H	75%

## Data Availability

The raw sequencing data were deposited in the NCBI Sequence Read Archive (SRX21294582—Illumina sequencing data and SRX21294583–PacBio sequencing data). The Whole Genome Shotgun project has been deposited at DDBJ/ENA/GenBank under the accession JBCLNQ000000000. The version described in this paper is version JBCLNQ010000000.

## Supplementary Materials

The following supporting information can be downloaded at: https://www.mdpi.com/article/10.3390/jof10050352/s1, Figure S1: The ORF and amino acid translation of *DDC* gene in the genome of strain DC2; Table S1: Comparison analysis of genome features of strain DC2 and 75 available *Talaromyces* strain in NCBI.
