# Supplementary material for: Genome Characteristics of the Endophytic Fungus Talaromyces sp. DC2 Isolated from Catharanthus roseus (L.) G. Don
Source: J Fungi (Basel). 2024 May 15;10(5):352. doi: 10.3390/jof10050352 (PMC11122143; doi:10.3390/jof10050352)
Supplement: Supplementary file 1 [file jof-10-00352-s001.zip › jof-2925174-supplementary/Supplementary Table S1.pdf]

Supplementary Table S1. Comparison analysis of genome features of strain DC2 and 75 available *Talaromyces* strain in NCBI

No	Scientific name	Strains	Genome size	Assembly level	Data resource
1	*Talaromyces piceae*	9-3	26,564,319	Scaffold	GCA_001657655.1
2	*Talaromyces borbonicus*	SV-2017a	27,050,413	Scaffold	GCA_002916415.1
3	*Talaromyces marneffei*	WCHTM105701	27,182,151	Contig	GCA_006111635.1
4	*Talaromyces marneffei*	PUMCH_TM1901	27,617,670	Scaffold	GCA_011320185.1
5	*Talaromyces sp. P2101*	PF2101	27,704,390	Scaffold	GCA_023626015.1
6	*Talaromyces funiculosus*	PF3603	27,934,616	Scaffold	GCA_023623875.1
7	*Talaromyces funiculosus*	D2P3	28,135,311	Scaffold	GCA_023623855.1
8	*Talaromyces marneffei*	11CN-20-091	28,198,338	Complete	GCF_009556855.1
9	*Talaromyces marneffei*	11CN-03-130	28,216,733	Chromosome	GCA_009650675.1
10	*Talaromyces marneffei*	TM4	28,307,147	Complete	GCA_003971505.1
11	*Talaromyces marneffei*	GZ8H79	28,376,234	Contig	GCA_013122295.1
12	*Talaromyces funiculosus*	R1404	28,385,195	Scaffold	GCA_023623975.1
13	*Talaromyces funiculosus*	X33	28,488,493	Complete	GCA_004299765.1
14	*Talaromyces funiculosus*	PK3801	28,528,190	Scaffold	GCA_023624025.1
15	*Talaromyces marneffei ATCC 18224*	ATCC 18224	28,643,865	Scaffold	GCF_000001985.1
16	*Talaromyces funiculosus*	D2B1	28,724,527	Scaffold	GCA_025768105.1
17	*Talaromyces funiculosus*	R1212	28,858,126	Scaffold	GCA_023623845.1
18	*Talaromyces sp. PT2101*	PF3502	28,879,243	Scaffold	GCA_023626035.1
19	*Talaromyces marneffei PM1*	PM1	28,887,485	Contig	GCA_000227055.2
20	*Talaromyces marneffei PM1*	PM1	29,022,615	Contig	GCA_000750115.2
21	*Talaromyces funiculosus*	D1B1	29,039,825	Scaffold	GCA_023623995.1
22	*Talaromyces purpureogenus*	Q2	29,077,759	Contig	GCA_019022425.1
23	*Talaromyces funiculosus*	RG13M1	29,154,027	Scaffold	GCA_023623825.1
24	*Talaromyces sp. PYS2103*	P2101	29,277,153	Scaffold	GCA_023625975.1
25	*Talaromyces sp. PH2104*	PT2101	29,383,702	Scaffold	GCA_023625955.1
26	*Talaromyces sp. F3606*	PYS2103	29,457,564	Scaffold	GCA_023625895.1
27	*Talaromyces funiculosus*	R2203A	29,504,766	Scaffold	GCA_023623985.1
28	*Talaromyces ruber*	FKI-L3-BK-DAB3	30,034,193	Scaffold	GCA_022813215.1
29	*Talaromyces atroroseus*	IBT 11181	30,858,562	Scaffold	GCF_001907595.1
30	*Talaromyces thailandensis*	OC-R06-P5	31,813,847	Scaffold	GCA_019828575.1
31	*Talaromyces trachyspermus*	4014	32,038,095	Contig	GCA_020137715.1
32	*Talaromyces sp. PL2406*	B14P1	32,900,204	Scaffold	GCA_023626215.1
33	*Talaromyces sp. PT3601*	M1327	33,633,690	Scaffold	GCA_023626395.1
34	*Talaromyces sp. R2412*	PX1902	33,681,611	Scaffold	GCA_023626135.1
35	*Talaromyces amestolkiae*	CIB	33,721,883	Scaffold	GCF_001896365.1
36	*Talaromyces amestolkiae*	X1601	33,824,912	Scaffold	GCA_023623795.1
37	*Talaromyces sp. PF2101*	X2403	33,878,408	Scaffold	GCA_023626055.1
38	*Talaromyces amestolkiae*	G173	33,972,799	Contig	GCA_027569865.1
39	*Talaromyces sp. R2405*	R2412	34,240,689	Scaffold	GCA_023626155.1
40	*Talaromyces sp. PX2406*	PL2406	34,271,254	Scaffold	GCA_023626115.1
41	*Talaromyces sp. B14P1*	PYS2902	34,461,311	Scaffold	GCA_023626195.1
42	*Talaromyces stollii*	CLY-6	34,462,933	Scaffold	GCA_014065225.1
**43**	***Talaromyces radicus***	**DC2**	**34,575,287**	**Contig**	**This study**
44	*Talaromyces sp. PX2802*	PF30-10	34,594,379	Scaffold	GCA_023626255.1

45	*Talaromyces sp. PF3502*	PM2405	34,671,233	Scaffold	GCA_023625995.1
46	*Talaromyces sp. PF30-1C*	PF30-5	34,714,567	Scaffold	GCA_023626275.1
47	*Talaromyces islandicus*	WF-38-12	34,715,840	Scaffold	GCA_000985935.1
48	*Talaromyces sp. PF30-10*	PF30-1C	34,746,589	Scaffold	GCA_023626295.1
49	*Talaromyces sp. PG4302*	PT3601	34,770,149	Scaffold	GCA_023626335.1
50	*Talaromyces pinophilus*	PSF-12(1)	34,770,673	Contig	GCA_003316675.1
51	*Talaromyces sp. PYS2902*	PF30-1D	34,806,200	Scaffold	GCA_023626235.1
52	*Talaromyces sp. PF30-1D*	PG4302	34,879,448	Scaffold	GCA_023626315.1
53	*Talaromyces sp. PX1902*	PX2802	35,032,352	Scaffold	GCA_023626175.1
54	*Talaromyces sp. X2403*	PX2406	35,045,590	Scaffold	GCA_023626095.1
55	*Talaromyces sp. PM2405*	R2405	35,331,725	Scaffold	GCA_023626075.1
56	*Talaromyces sp. PF30-5*	S1602	35,401,712	Scaffold	GCA_023626375.1
57	*Talaromyces liani*	FKII-L2-CM-P1	35,494,196	Scaffold	GCA_022814625.1
58	*Talaromyces liani*	FKII-L2-CM-DRAB3	35,499,968	Scaffold	GCA_022814505.1
59	*Talaromyces stipitatus ATCC 10500*	ATCC 10500	35,685,443	Scaffold	GCF_000003125.1
60	*Talaromyces rugulosus*	W13939	35,758,560	Complete	GCF_013368755.1
61	*Talaromyces adpressus*	CBS 142503	36,107,696	Scaffold	GCA_002775195.1
62	*Talaromyces pinophilus*	AR155	36,366,659	Scaffold	GCA_011392495.1
63	*Talaromyces pinophilus*	Y-94	36,402,801	Scaffold	GCA_000829775.1
64	*Talaromyces pinophilus*	1-95	36,480,443	Complete	GCA_001571465.2
65	*Talaromyces pinophilus*	J7Y3	36,811,154	Scaffold	GCA_023623735.1
66	*Talaromyces pinophilus*	M8004A	37,375,402	Scaffold	GCA_023623785.1
67	*Talaromyces pinophilus*	P8041	37,439,112	Scaffold	GCA_023623755.1
68	*Talaromyces pinophilus*	NRRL 3647	37,575,941	Contig	GCA_027569565.1
69	*Talaromyces pinophilus*	NRRL 3503	37,579,131	Contig	GCA_027569545.1
70	*Talaromyces proteolyticus*	PMI_201	37,611,490	Contig	GCF_021365285.1
71	*Talaromyces verruculosus*	TS63-9	37,629,238	Scaffold	GCA_001305275.1
72	*Talaromyces albobiverticillius*	Tp-2	38,354,882	Contig	GCA_023721895.2
73	*Talaromyces pinophilus*	NA01	38,535,829	Contig	GCA_009805475.2
74	*Talaromyces wortmannii*	LMB-HP14	38,588,669	Scaffold	GCA_001939245.1
75	*Talaromyces purpureogenus*	MYA-38	38,853,631	Scaffold	GCA_001270325.1
76	*Talaromyces nanjingensis*	JP-NJ4	42,526,372	Contig	GCA_031010415.1
