# Supplementary figures and images for: Genome Characteristics of the Endophytic Fungus Talaromyces sp. DC2 Isolated from Catharanthus roseus (L.) G. Don
Source: J Fungi (Basel). 2024 May 15;10(5):352. doi: 10.3390/jof10050352 (PMC11122143; doi:10.3390/jof10050352)

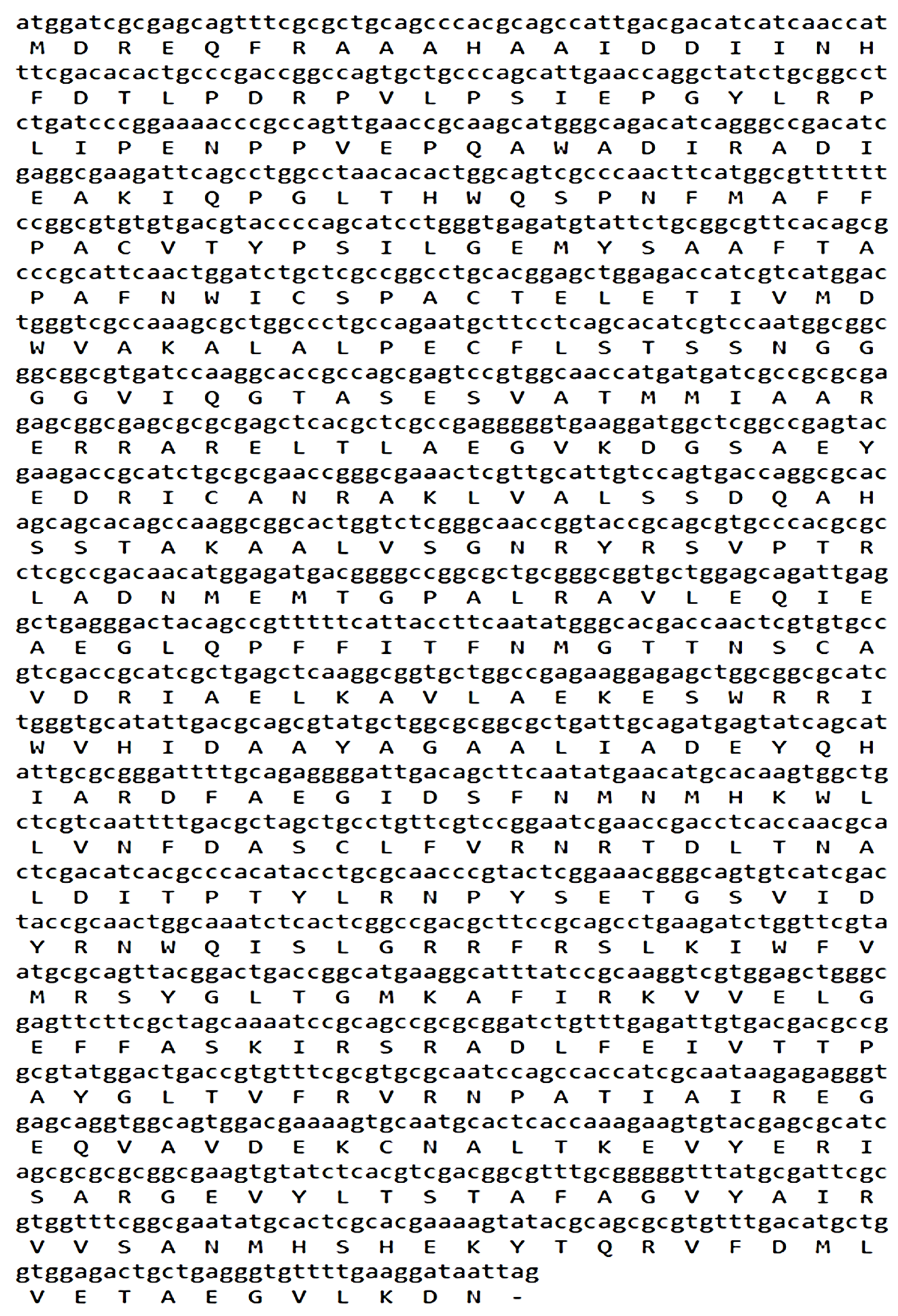

Supplement: Supplementary file 1 [file jof-10-00352-s001.zip › jof-2925174-supplementary/Supplementary Figure S1.tif]
